# A New Perspective on Metabolic Syndrome with Osteopontin: A Comprehensive Review

**DOI:** 10.3390/life13071608

**Published:** 2023-07-22

**Authors:** Ipek Cicekli, Duygu Saglam, Nadir Takar

**Affiliations:** 1 Department of Nutrition and Dietetics, Faculty of Health Sciences, Acibadem Mehmet Ali Aydinlar University, Istanbul 34752, Turkey; duygu.saglam@acibadem.edu.tr; 2 Department of Family Medicine, Kartal Dr. Lutfi Kirdar City Hospital, Istanbul Provincial Directorate of Health, Istanbul 34865, Turkey; nadirtakar@gmail.com

**Keywords:** metabolic syndrome, osteopontin, diabetes, obesity, atherosclerosis, hypertension, risk factors

## Abstract

Metabolic syndrome (MetS) imposes a substantial burden on the healthcare systems and economies of countries and is a major public health concern worldwide. MetS is mainly caused by an imbalance between calorie intake and energy expenditure; however, it is recognized that additional variables, such as chronic inflammation, may have the same predictive potential as insulin resistance or MetS components in the genesis of type 2 diabetes and cardiovascular events. More importantly, the early diagnosis or treatment of MetS may significantly reduce the burden on the health systems of the disease with any prevention or biomarker and should not be underestimated. Osteopontin (OPN), also called secreted phosphoprotein 1, is a soluble protein found mostly in body fluids. Studies suggest that serum OPN levels may be an early and new biomarker to predict metabolic and cardiovascular complications significantly associated with some diseases. This review aims to provide specific insight into the new biomarker OPN in MetS. With this purpose, it is examined the link between the MetS cornerstones and OPN. In addition, the interaction between the microbiota and MetS is predicted to be bidirectional, and the microbiota may act as a bridge in this interaction process. Increased OPN levels may have unfavourable consequences for cardiovascular diseases, diabetes, and obesity, all of which are components of MetS. Further studies are required to evaluate the use of OPN levels as a clinical biomarker risk of MetS.

## 1. Introduction

Non-communicable diseases (NCDs) have become the leading causes of morbidity and mortality not only in developed countries but also in less developed countries [1]. NCDs cause greater increases in morbidity and mortality, lower quality of life, and increased public health care spending, particularly in low- and middle-income countries [2]. One of these NCDs, metabolic syndrome (MetS), imposes a substantial burden on the healthcare systems and economies of countries and is a major public health concern worldwide [1].

MetS is mainly caused by an imbalance between calorie intake and energy expenditure [1]. MetS is diagnosed in the presence of at least three of these five risk factors: central obesity, fasting plasma glucose level ≥ 5.6 mmol/L, triglyceride level ≥ 1.7 mmol/L, HDL cholesterol level < 1.03 mmol/L in men and < 1.29 mmol/L in women, and elevated blood pressure [3]. However, it is also a complex pathophysiological process influenced by factors such as the genetic/epigenetic structure of the individual, a lifestyle with lack of movement, a quality and balanced diet, and intestinal microbiota. Unfortunately, it has become a global epidemic and there is no obvious solution to ending or mitigating this situation. The epidemic was not unforeseeable and could not be effectively contained; it can only be managed if there is a social will at that. As with other outbreaks, it will be crucial to inform and give education to individuals about the health risks of MetS [1].

A study in adults from 2007 to 2012 reported the prevalence of MetS to be 33% by 2016. Nonetheless, the prevalence of MetS increases significantly with increasing age in all groups. While the prevalence is 19.5% in those aged 20–39, it rises to 48.6% in those aged at least 60 years. There was no significant difference in the prevalence of MetS between men and women in all age groups [4]. Another study reported that factors that were significantly associated with MetS in the general population were hypertension, high BMI, diabetes, and moderate-intensity physical activity. Diabetes and hypertension were shown to be related with MetS in males when gender-specific risks were examined using regression models, whereas high BMI, diabetes, and hyperlipidemia were associated with MetS in women [5]. However, it is recognized that additional variables, such as chronic inflammation, may have the same predictive potentials as insulin resistance or MetS components in the genesis of type 2 diabetes and cardiovascular events. More importantly, the early diagnosis or treatment of the metabolic syndrome should not be underestimated and significantly reduce the burden on the health systems of the disease with any prevention or biomarker.

Osteopontin (OPN), also called secreted phosphoprotein 1, is a soluble protein found mostly in bodily fluids [6]. Studies suggest that serum OPN levels may be an early and new biomarker to predict metabolic and cardiovascular complications significantly associated with cardiovascular diseases and diabetes mellitus [7]. Studies that directly address the relationship between OPN levels and MetS or its early effects in people with the disease are insufficient and studies often have been conducted on the components of MetS.

This review aims to provide specific insight into the new biomarker OPN in MetS. The cornerstones of MetS were determined as the four diseases of atherosclerosis, hypertension, obesity, and diabetes, by examining the literature, and this review is based on these categories. The connection between MetS cornerstones and OPN has been examined in the microbiota axis and it is predicted that it will act as a bridge in the bidirectional interaction between them.

## 2. Metabolic Syndrome and Osteopontin

OPN, also called secreted phosphoprotein 1, is a soluble protein found mostly in bodily fluids [6]. For instance, the brain, the ganglia in the inner ear, saliva, the dentin layer of the tooth, salivary and sudoriferous glands, milk, mammary gland cells, kidney, bile, endothelial cells, bone marrow, skeletal muscle cells, smooth muscle cells, decidua layer of the uterus, chorionic villus, pancreatic, and bile tracts [8]. Despite the fact that OPN can exist intracellularly as a regulator of cytoskeletal dynamics and gene expression, a large portion of the studies has focused on the secreted form [6]. In addition, OPN in bone tissue is produced by osteoblasts and osteoclasts, which are involved in osseous formation and resorption processes. Nonetheless, OPN generated by osteoclasts is known to block hydroxyapatite crystals, demonstrating that OPN is associated with bone disintegration [6]. Studies suggest that serum OPN levels may be an early and new biomarker to predict metabolic and cardiovascular complications significantly associated with cardiovascular diseases and diabetes mellitus [7]. 

Several studies illustrate that secretion of OPN is influenced by various dietary components. A phosphorus-enriched diet increases the expression of OPN in the renal tubules. With a high phosphorus diet, OPN in the renal tubules may act as a matrix component during the production of Ca deposits [9]. Moreover, studies claim that some diets boost OPN secretion, while others claim that specific foods decrease OPN secretion. For example, replacing saturated fat with unsaturated fat in a high-fat diet or reducing dietary fat attenuates obesity-associated inflammation and insulin resistance by reducing macrophage infiltration and the upregulation of OPN into adipose tissue induced through a high-fat diet [10]. In one study investigating the effect of dietary casein and whey on energy balance, the morbidity associated with stroke and kidney injury in salt-laden and high-fat-fed mice found that both casein and whey prevented stroke-related morbidity and reduced serum OPN levels [11].

Table 1 summarizes OPN levels and expressions associated with the MetS cornerstones in the review. 

### 2.1. Cornerstone 1: Atherosclerosis and Osteopontin

OPN is highly secreted from foam and macrophage cells of atherosclerotic plaques. In the clinical idea, OPN has been found to be related to numerous inflammatory diseases, including cardiovascular burden [34]. Due to the lower serum plasma level of OPN providing protection against post-myocardial infarction (MI) left ventricular dilation (by activating downstream signalling pathways such as mitogen-activated protein kinase, extracellular signal-regulated kinase, c-Jun N-terminal kinases, and the PI3K/Akt pathway) [35], it is thus crucial to dissect OPN anti-calcific and pro-inflammatory functions [34].

Calcification of the vessels, the most evident and dangerous sign of atherosclerosis, is a common occurrence in people with coronary artery disease (CAD) and vascular calcification is thought to be connected with an increased risk of MI [35]. According to a case-control study, OPN increases the risk of arterial calcification and the development of atherosclerotic plaques in arteries. Consequently, along with the induction of soft tissue biomineralization like the vascular wall, high serum levels of OPN have also been associated with CAD [13]. A study hypothesized that plasma levels of OPN, a new potential biomarker, are raised in patients with congestive heart failure and so this rise is also linked with disease projection and severity [12]. On the other hand, higher expression levels of OPN are found in vascular smooth muscle cells (VSMCs) in atherosclerotic plaques. In particular, apoptosis and VSMC migration are crucial markers of atherosclerosis progression. VSMC targeting strategies represent an effective method for discriminating delivery of anti-atherosclerotic drugs. The dominating role of OPN here is the ability of increasing inflammation in the atherosclerotic plaque and restricting vascular calcification which is involved in the atherosclerosis process. Furthermore, it has been shown that OPN correlated with the severity of CAD [14]. In another cardiac condition, heart failure, the role of OPN in calcification contributes to increased vascular resistance and ultimately to the development of disease [16]. As is already known, chronic congestive heart failure is a serious public health issue that is on the rise and with successful care relies on prompt and precise diagnoses, as well as simple risk stratification to identify patients who are most at risk of decompensation and death [12]. Despite the role of OPN in atherosclerosis-related pathways, little research has investigated whether OPN levels are linked to poor cardiovascular outcomes in individuals with established cardiovascular disease in this risk classification. As a matter of fact, plasma OPN levels have been independently linked with the composite incident endpoint of poor cardiovascular events (aOR: 2.04 1.44, 2.89) as well as incident hospitalization for heart failure (aOR: 2.04 1.44, 2.89) in patients with stable CAD [16]. Furthermore, by directing immunological response and VSMC migration, OPN has also been linked to atherosclerosis, neointima and plaque development, and dystrophic calcification. While enhanced OPN expression has the ability to defend against vascularization, it can also promote cardiac fibrosis and induce pathological remodelling through an increased extracellular matrix, collagen production, and deposition. This appears to be achieved by the stimulation of downstream signalling pathways [34]. Therefore, OPN levels may be an important therapeutic target in the atherosclerotic process. Serum OPN levels have been shown to be associated with cardiovascular events, but studies need to clarify the cut-off point of this biomarker. There are several studies that explore this cut-off point. One study separated all of the enrolled participants into three groups, assuming equal numbers of subjects per tertile, according to their baseline OPN levels: <2604 pg/mL (Tertile 1), 2605–4809 pg/mL (Tertile 2), and >4810 pg/mL (Tertile 3). According to the aforementioned study, OPN was an independent predictor of adverse cardiac outcomes in patients with chronic coronary syndrome, and an elevated OPN level was associated with a higher risk of acute myocardial infarction-related hospitalization (aOR: 3.20 1.23–8.33), particularly in those with OPN levels greater than 4810 pg/mL (Tertile 3). In addition, the combination of OPN and high-sensitivity-CRP indicated an increase in cardiac events [15]. However, no reference value for OPN has been reported in normal healthy populations or patients with CAD. However, because the research included patients with chronic coronary syndrome who had already undergone percutaneous coronary intervention, OPN levels in the study sample might be significantly higher [15]. Given that OPN levels were considerably higher in patients with heart failure and linked with functional status, Rosenberg et al. (2008) conducted a cohort study to determine the ideal OPN plasma level for prospective prediction of mortality within 48 months of follow-up. As a result, the optimum OPN cut-off value for predicting all-cause mortality after 48 months was 929 ng/mL, with a sensitivity of 46% and a specificity of 83% [12]. As stated in the study, OPN plasma levels were considerably higher in patients with systolic heart failure. Furthermore, it is hypothesized that OPN plasma levels may be used to predict outcomes independently of recognized clinical and biochemical indicators, such as N-terminal prohormone brain natriuretic peptide [12]. These findings imply that OPN is a new marker protein involved in the cardiac response to biomechanical load and myocardial damage [12]. Nevertheless, the best OPN cut-off value for predicting cardiovascular disease outcomes could not be clarified in the studies. Further research into the exact mechanisms of the dysregulated rise of OPN in chronic coronary syndrome patients are needed [15]. 

### 2.2. Cornerstone 2: Hypertension and Osteopontin

Inflammation is an essential component of many diseases. Inflammatory biomarkers that strongly predict cardiovascular diseases may have the potential to improve planned treatment strategies. OPN, which is a secreted protein, has a special interest as a novel biomarker due to its measurably in body fluids including plasma, cerebrospinal fluid, breast milk, and urine. Due to OPN’s measurably with minimally invasive instruments, it provides the advantage of rapid and repeated measurement over time [36].

The vessel wall remodels in response to pulsatile flow and pressure increase. In hypertension, OPN expression is increased by a partial increase in aortic tension and an increase in reactive oxygen species production [17]. Activation of the phosphatidylinositol-3 kinase/Akt1 (known as protein kinase B) signalling pathway is one of the mechanisms by which mechanical strain increases OPN expression in VSMCs [18]. A peptide hormone Ang II that causes vasoconstriction and increases blood pressure, promotes hypertension and upregulates OPN expression particularly through Ang II in the production of reactive oxygen species [17]. 

OPN has been associated with hypertension-related inflammatory cell recruitment and vascular remodelling. Subsequently, the expressions of levels are significantly higher in aortic tissues and plasma in hypertensive rodents, and expression is positively correlated with systolic blood pressure [18] suggesting that OPN might be used as a new clinical marker for hypertension-induced vascular remodelling. One such study explains that treatment with statins and Ang II blockers significantly reduces plasma OPN levels [20]. Serum OPN levels are also favorably linked with arterial stiffness and significantly raised in arterial hypertension due to inflammatory factors that are amplified [13,19]. However, whether OPN affects peripheral monocyte differentiation and expression of inflammatory factors in hypertensive individuals with vascular calcification remains still unclear.

Ge et al. (2017) investigated if OPN regulates macrophage activation and osteoclast development in hypertensive patients with vascular calcification and it has been suggested that OPN-mediated macrophage activation plays a potential role in the regulation of hypertension-associated vascular calcification. Hypertensive patients with vascular calcification are characterized by a significantly increased peripheral proinflammatory monocyte ratio and increased serum OPN level when compared to hypertensive patients without vascular calcification. Most notably, the study shows that OPN modulates monocyte/macrophage phenotypic differentiation in hypertensive individuals with vascular calcification including attenuation of macrophage-osteoclast differentiation and inhibition of expression of inflammatory factors [21]. 

Stepien et al. (2011) found significant correlations between OPN levels and the inflammation marker c-reactive protein, but did not observe any correlation between fibrinogen and OPN. The study highlights the very strong association between hypertension and OPN levels (above 52 ng/mL). Inflammatory processes play key roles in endothelial dysfunction between hypertension and OPN levels. Interestingly, in the study it is also shown that risk factors such as gender, age, and diabetes mellitus had no significant effect of hypertension on raising OPN levels [19]. Additionally, a positive link between fasting glucose levels and OPN suggests that insulin resistance may upregulate the inflammatory process resulting in elevated CRP levels and impaired vascular function. Although CRP has not been identified as a determinant of serum OPN levels, it can be hypothesized that inflammation is a possible mechanism of endothelial dysfunction causing hypertension. The optimal cut-off point for OPN was determined to be 19.7 ng/mL to distinguish between hypertensive and asymptomatic subjects; however, the sensitivity and specificity of these tests are insufficient to employ OPN as the primary constructive tool for identifying endothelium impairment [19].

### 2.3. Cornerstone 3: Diabetes and Osteopontin

OPN is a multifunctional pro-inflammatory cytokine that is expressed in various cell types and tissues and involved in many physiologic and pathological processes. It has been established as a major component in the development of adipose tissue inflammation and insulin resistance and also it is thought that it may play a role in the pathogenesis of diabetes [37]. 

Pro-inflammatory cytokines, which play an important role in the development of diabetes complications, rise in response to OPN release [22]. The pathway is due to the fact that it is a multifunctional molecule that is selectively expressed in surrounding inflammatory cells in chronic inflammatory and autoimmune disorders. Furthermore, this biomarker is a secreted sticky molecule that is considered to help in monocyte-macrophage recruitment and control cytokine production in macrophages, dendritic cells, and T-cells. Therefore, OPN modulation of immune cell response has been linked to a variety of inflammatory conditions and may be crucial in the development of adipose tissue inflammation and insulin resistance [38]. In a study, high correlations of OPN levels with beta-cell function demonstrates the link between the inflammatory score and type 2 DM. A higher inflammatory score is associated with impaired beta-cell function, which is consistent with several studies that have shown that the histology of islets from patients with type 2 DM shows typical features of tissue inflammation, such as higher expression of cytokines and chemokines, immune cell infiltration, decreased insulin staining, cell apoptosis, and islet amyloidosis [39]. Another study discovered that plasma OPN levels correlated with the severity of diabetic nephropathy and CAD, implying that an elevated plasma OPN level might be utilized as a biomarker for screening diabetic vasculopathy [22]. As a result, it has been suggested that OPN plays an important role in the development of insulin resistance by enhancing macrophage accumulation in adipose tissue and encouraging inflammatory creation. These findings imply that OPN may play an important role in the development of insulin resistance by increasing inflammation and the recruitment of macrophages in adipose tissue [26].

Reducing circulating OPN levels is linked to improvements in blood pressure, LDL-c, HDL-c, and glycemic control. In a one-year follow-up study, serum OPN levels were found to be independent predictors of glycemic profile improvement. Glycemic improvement was seen in individuals who also reduced their circulating OPN levels. The study, thus, supposed that higher OPN levels at baseline indicate glycemic profile stabilization [23]. OPN has also been linked with diabetic retinopathy and nephropathy in patients with type 2 diabetes [27]. 

Daniele et al. suggested that plasma OPN levels in patients with type 2 DM were greater than in the control group in research on patients with type 2 DM and healthy controls. Another finding was a link between plasma OPN levels and tumor necrosis factor secretion, which mediates obesity-induced insulin resistance. This study reveals that hyperglycemia is also closely connected to the inflammatory state in type 2 diabetes. OPN might enhance the detection of low-grade inflammation in obese mice and patients with type 2 DM, as well as the prediction of abnormalities in glucose metabolism [24]. Although there are studies which have established a relationship between OPN levels and type 2 DM [24,27] and insulin resistance [28], studies on type 1 DM are insufficient. In the pediatric population with type 1 diabetes, elevated levels of OPN are related to poor metabolic control as evaluated by glycated hemoglobin and preclinical atherosclerosis [25]. In another study conducted with adults with type 1 diabetes, serum OPN is a robust predictor of incipient diabetic nephropathy and all-cause death [25]. These findings imply that OPN may play an important role in the development of insulin resistance by increasing inflammation and the recruitment of macrophages in adipose tissue [26]. However, more studies are needed to elucidate its effect on the pathogenesis of diabetes, especially type 1 DM. The fact that there are very few studies with type 1 DM indicates that there is a significant gap in the literature in this area. 

Studies showed that serum OPN is independently associated with MetS. Serum OPN can be potentially used as a biochemical parameter for risk stratification of MetS [37]. 

### 2.4. Cornerstone 4: Obesity and Osteopontin

Low-grade inflammation, which is associated with an increase of macrophages and cytokines in the tissue, is a characteristic of adipose tissue growth in obesity. Furthermore, low-grade inflammation may be a contributing factor to the relationship between obesity and systemic insulin resistance. Adipokines are cytokines that are created and present in adipose tissue and include leptin, resistin, IL6, OPN, and others. OPN is one of the most prevalent cytokines in people with obesity adipose tissue [26]. In one study, mice given a high-fat diet became obese and had higher circulating OPN concentrations, while obese animals without OPN had higher insulin sensitivity, indicating that OPN is a major factor in high-fat diet-induced insulin resistance [26]. Furthermore, OPN deficiency not only leads to declined adipose tissue inflammation but also recovers reduced insulin resistance and glucose tolerance in mice independent of energy expenditure or body composition [29]. 

Chapman et al. (2010) showed that OPN depletion protects against metabolic impairment after only two weeks of feeding with a high-fat diet [28]. Despite a higher caloric intake, OPN depletion prevents the increase in body weight and adipose tissue expansion caused by a high-fat diet, as well as decreasing macrophage inflammation, infiltration, fibrosis, and oxidative stress [30].

Gómez-Ambrosi et al. (2007) highlighted that plasma OPN concentrations are significantly higher in people who are overweight or with obesity and circulating OPN concentrations correlate with body fat. Moreover, OPN mRNA and protein are expressed in omental adipose tissue and this expression is increased in obesity and further elevated in obesity-associated type 2 DM. Finally, modest diet-induced weight loss is accompanied by a significant decrease in plasma OPN levels [31]. In the aforementioned study, patients with obesity had a two-fold rise in plasma OPN concentrations compared to lean people. A substantial positive association was demonstrated in the study between OPN and body fat, indicating that OPN levels are connected to the quantity of adipose tissue [31]. The decrease in OPN plasma concentrations reported in people with obesity following weight loss may contribute to a lower cardiovascular risk profile. Therefore, a drop in OPN levels may contribute to the decrease in cardiovascular morbidity reported following weight loss [31].

As a consequence of morbid obesity, bariatric surgery procedure is an important route. Studies examining OPN levels in bariatric surgery patients discovered surprising findings in severely obese patients before and after bariatric surgery. Subjects who had previously had gastric banding or Roux-en-Y gastric bypass had lower body weight, BMI, inflammatory markers, and insulin resistance. All studies, however, uniformly documented a steady rise in OPN blood concentrations following bariatric surgery [32,33].

Consequently, results show that monocytes and macrophages not only respond to OPN and migrate to areas of inflammation, but they also survive and multiply more in the presence of OPN, confirming the established mechanism. Subsequently, secreted OPN appears to be a crucial participant in inflammation, not only by promoting monocyte chemotaxis and macrophage differentiation but also by promoting macrophage local proliferation [40]. As a result of the relationship between obesity and diabetes, obesity is now recognized to contribute to insulin resistance through persistent low-grade inflammation arising from visceral adipose tissue, which raises the chance of developing diabetes. Early identification of serum circulating molecules, particularly OPN, might be a promising technique for early diagnosis and, ultimately, preventing or delaying both diabetes and obesity effects [7].

## 3. Can Microbiota Serve as a Bridge?

**Figure 1:**
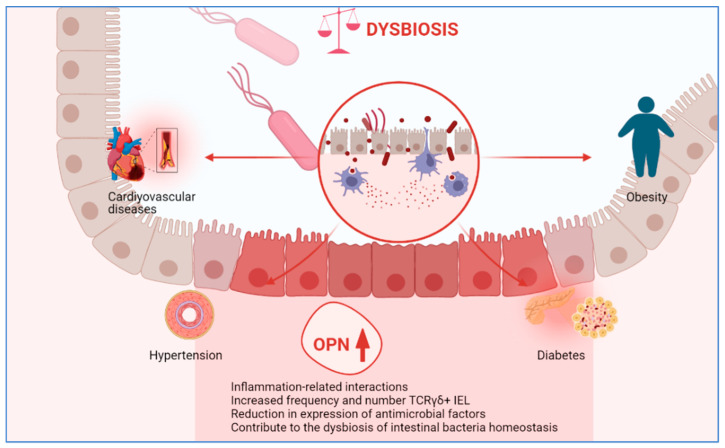
Possible associations between OPN and gut microbiota.

The gut microbiota play a key role in maintaining the physiological function of the host, and dysbiosis of the gut microbiota caused by many factors leads to significant physiological changes and increases the risk of MetS [41]. It has been suggested that gut microbiota dysbiosis has a strong link with diabetes [42,43], cardiovascular diseases [44,45], dyslipidemia [46], obesity [47], and hypertension [48], which are components of MetS. The possible relationship of OPN with metabolic syndrome cornerstones in gut microbiota is summarized in Figure 1.

Recent evidence indicates that the gut microbiota is one of the most important and modifiable pathogenic factors in MetS. MetS is often accompanied by an imbalance of the gut microbiota and breaks down the gut barrier, causing a low-grade inflammatory response in the body. Then, by affecting host metabolism and hormone secretion via metabolites, it causes insulin resistance and creates a continuous vicious circle. Therefore, the gut microbiota may be a potential target in the treatment of MetS [41]. 

It has been demonstrated by immunohistochemistry that OPN is distributed on epithelial cells and plasma cells in normal epithelial tissue [49]. Several groups reported that OPN is involved in inflammatory bowel diseases including Crohn’s disease and ulcerative colitis, which are caused by excessive responses to commensal microbiota and other intestinal antigens [50].

Inflammation-related interactions of OPN have been frequently studied in the literature, but the effect of OPN on the healthy gut is still unclear. Tissue cells in the gut are constantly exposed to antigens from the microbiota, and OPN expression increases in response to some infections [51]. Thus, it has been hypothesized that OPN might be induced in immune cells by exposure to antigens from these microbes.

A study investigating the role of OPN in normal intestinal tissues used reporter mice in which the OPN gene was replaced by EGFP (EGFP-OPN knock-in mice) to monitor OPN expression. Particularly, the study found a reduction of frequency and number in T cell receptor γδ+ intraepithelial lymphocytes (TCRγδ+ IEL) in OPN-deficient mice and the microbiota of faecal samples was dramatically altered compared with wild type mice. Thus, the study suggests the possibility that OPN may contribute to the maintenance of intestinal commensal bacteria homeostasis, possibly by TCRγδ+ IEL survival. Also, the study shows that OPN has a significant effect on the expression of antimicrobial factors in intestinal tissue. Therefore, serum OPN level may be associated with obesity and inflammatory diseases, including MetS. Further studies with plasma levels of OPN, where this mechanism is explained, is highly recommended [52]. 

## 4. Conclusions

MetS has reached alarming levels all over the world in all age groups, including children and adolescents. Therefore, early diagnosis and each step of progress in treatment can be predictive in the prevention of the disease. In the large window created by the combination of the cornerstones mentioned in this study, OPN levels may be an important biomarker in improving global public health. 

The most effective approach to predicting MetS is to use a combination of clinical and laboratory indicators and take into account individual risk variables such as lifestyle factors and family history. Predicting MetS is only the first step; preventing or treating the disease requires a comprehensive approach that includes lifestyle changes, regular monitoring, and medications [53]. However, an increasing body of research demonstrates that OPN overexpression is critical in controlling compensatory heart fibrosis and hypertrophy; however, there are very few studies that can directly establish a relationship between OPN and MetS. 

Increased OPN levels may have unfavourable consequences such as cardiovascular disease, diabetes, and obesity, all of which are components of MetS. Further studies are required to evaluate the use of OPN levels as a clinical biomarker. In order to better understand the effect of OPN on MetS, there is a need for well-designed human studies that can be performed at all levels of the disease.

## Figures and Tables

**Table 1:** Associations of higher OPN levels and depletion with atherosclerosis, hypertension, diabetes, and obesity.

Cornerstones	Author	Year	Study Population	Main Findings
Atherosclerosis	Rosenberg et al. [12]	2008	*n* = 420 patients with chronic heart failure	Higher levels in patients with heart failure and linked with functional status
	Tousoulis et al. [13]	2013	*n* = 409 patients undergoing diagnostic coronary angiography	Increases the risk of CAD
	Berezin et al. [14]	2013	*n* = 126 patients (46 patients with type 2 DM) with asymptomatic CAD	Increases the risk of arterial calcification
				Increases development of atherosclerotic plaques in arteries
				Higher expression levels of OPN in VSMCs in atherosclerotic plaques
	Cheong et al. [15]	2023	*n* = 948 participants	A higher risk of acute myocardial infarction-related hospitalization
	Abdalrhim et al. [16]	2016	*n* = 3567 CAD patients	Higher OPN levels associated with a higher incidence of heart failure hospitalization
Hypertension	Caesar et al. [17]	2017	*n* = 6 OPN-KO and wild type mice	Increased expression of OPN by a partial increase in aortic tension
				An increase in reactive oxygen species production
	Seo et al. [18]	2015	Hypertensive mice	Associated with hypertension-related inflammatory cell recruitment and vascular remodelling
	Stepień et al. [19]	2011	*n* = 130 asymptomatic and hypertensive subjects	Linked with arterial stiffness
				Regulates macrophage activation and osteoclast development in hypertensive patients with VC
				Significantly raised in arterial hypertension
	Lorenzen et al. [20]	2010	*n* = 190 Adults with essential hypertension	Higher baseline OPN concentrations compared to healthy controls
	Ge et al. [21]	2017	*n* = 70 hypertensive patients	An increased serum OPN level is involved in monocytes/macrophage activation in hypertensive patients with VC
Diabetes	Yan et al. [22]	2010	*n* = 301 diabetic patients	Correlates with severity of diabetic nephropathy
	Caserza et al. [23]	2021	MetS patients (*n*= 85)	Reducing OPN levels is linked to improvements in glycemic control
	Daniele et al. [24]	2014	Type 2 DM patients and control group (*n* = 32)	Enhances the detection of low-grade inflammation in type 2 DM
	Abo El-Asrar et al. [25]	2018	*n* = 80 patients with type 1 DM	Related to poor metabolic control in children with type 1 DM
	Nomiyama et al. [26]	2007	*n* = 15 murine model of diet-induced obesity	OPN deficiency improves insulin sensitivity
	Yamaguchi et al. [27]	2004	*n* = 229 patients with type 2 DM	Plasma OPN levels increased significantly with ageing and the progression of diabetic nephropathy
	Chapman et al. [28]	2010	*n* = 13 male mice	OPN-KO mice are protected from HFD-induced insulin resistance
Obesity	Kiefer et al. [29]	2010	*n* = 13 male mice	OPN deficiency leads to declined adipose tissue inflammation
	Lancha et al. [30]	2014	*n* = 36 male wild type and OPN-KO	OPN depletion prevents the increase in body weight and adipose tissue expansion caused by HFD
	Gómez-Ambrosi et al. [31]	2007	*n* = 77 healthy volunteers (26 lean, 14 overweight and 37 obese)	Correlated with body fat
				Weight loss is accompanied by significant decreases in plasma OPN levels
				Decreases in OPN plasma concentrations contribute to a lower cardiovascular risk profile in people with obesity
	Riedl et al. [32]	2008	*n* = 40 patients with morbid obesity	Plasma OPN levels significantly increased after bariatric surgery and correlated with biomarkers of bone turnover
	Komorowski et al. [33]	2011	*n* = 28 patients with simple obesity and the presence of metabolic syndrome	A steady rise in OPN blood concentrations following bariatric surgery
	Wang et al. [10]	2013	*n* = 100 obese rats	Reducing dietary fat can attenuate the upregulation of OPN by a HFD
	Nomiyama et al. [26]	2007	*n* = 15 murine model of diet-induced obesity	OPN-KO mice are protected from obesity-associated adipose tissue and systemic inflammation
				OPN has no effect on the development of obesity and does not affect food intake or energy metabolism in mice with diet-induced obesity

OPN: osteopontin; DM: diabetes mellitus; CAD: chronic artery disease; VSMC: vascular smooth muscle cell; VC: vascular calcification; OPN-KO: osteopontin knockout; HFD: high fat diet.

## Data Availability

Data sharing is not applicable.
